# Silver and Carbon Nanomaterials/Nanocomplexes as Safe and Effective ACE2-S Binding Blockers on Human Skin Cell Lines

**DOI:** 10.3390/molecules29153581

**Published:** 2024-07-29

**Authors:** Anna Hotowy, Barbara Strojny-Cieślak, Agnieszka Ostrowska, Marlena Zielińska-Górska, Marta Kutwin, Mateusz Wierzbicki, Malwina Sosnowska, Sławomir Jaworski, André Chwalibóg, Ireneusz Kotela, Ewa Sawosz Chwalibóg

**Affiliations:** 1 Department of Nanobiotechnology, Warsaw University of Life Sciences, 02-786 Warsaw, Poland; barbara_strojny-cieslak@sggw.edu.pl (B.S.-C.); agnieszka_ostrowska@sggw.edu.pl (A.O.); marlena_zielinska-gorska@sggw.edu.pl (M.Z.-G.); marta_prasek@sggw.edu.pl (M.K.); mateusz_wierzbicki@sggw.edu.pl (M.W.); malwina_sosnowska@sggw.edu.pl (M.S.); slawomir_jaworski@sggw.edu.pl (S.J.); ewa_sawosz@sggw.edu.pl (E.S.C.); 2 Section of Production, Nutrition and Health, Department of Veterinary and Animal Sciences, University of Copenhagen, DK-1870 Frederiksberg, Denmark; 3 Department of Orthopaedics, National Medical Institute of the Ministry of the Interior and Administration, 02-507 Warsaw, Poland; ikotela@op.pl; 4 Collegium Medicum, Jan Kochanowski University in Kielce, 25-369 Kielce, Poland

**Keywords:** silver nanoparticles, diamond nanoparticles, graphene oxide, ACE2, SARS-CoV-2, HEKa, HDFa

## Abstract

(1) Background: Angiotensin-converting enzyme 2 (ACE2) is a crucial functional receptor of the SARS-CoV-2 virus. Although the scale of infections is no longer at pandemic levels, there are still fatal cases. The potential of the virus to infect the skin raises questions about new preventive measures. In the context of anti-SARS-CoV-2 applications, the interactions of antimicrobial nanomaterials (silver, Ag; diamond, D; graphene oxide, GO and their complexes) were examined to assess their ability to affect whether ACE2 binds with the virus. (2) Methods: ACE2 inhibition competitive tests and in vitro treatments of primary human adult epidermal keratinocytes (HEKa) and primary human adult dermal fibroblasts (HDFa) were performed to assess the blocking capacity of nanomaterials/nanocomplexes and their toxicity to cells. (3) Results: The nanocomplexes exerted a synergistic effect compared to individual nanomaterials. HEKa cells were more sensitive than HDFa cells to Ag treatments and high concentrations of GO. Cytotoxic effects were not observed with D. In the complexes, both carbonic nanomaterials had a soothing effect against Ag. (4) Conclusions: The Ag5D10 and Ag5GO10 nanocomplexes seem to be most effective and safe for skin applications to combat SARS-CoV-2 infection by blocking ACE2-S binding. These nanocomplexes should be evaluated through prolonged in vivo exposure. The expected low specificity enables wider applications.

## 1. Introduction

Angiotensin-converting enzyme 2 (ACE2) is a component of the renin–angiotensin system (RAS). Evidence shows that ACE2 provides protective effects in peripheral tissues and has great potential for treating RAS-related diseases [1]. Tightly controlled RAS activity is critical for maintaining the systemic hemodynamic and blood volume, and controlling cell proliferation, differentiation, and tissue remodelling in target organs. Endogenous RAS maintains the self-renewal and regeneration potential of epidermal stem cells (ESCs), thereby contributing to wound healing [2].

During the COVID-19 pandemic caused by the novel coronavirus SARS-CoV-2, the ACE2 enzyme was proven to be a crucial functional receptor of SARS-CoV-2 [3]. A prominent feature of ACE2 is the deep channel on the top of the molecule that contains the catalytic site. Negatively charged ridges surrounding the channel provide a binding site for the positively charged receptor-binding domain (RBD) of the S-glycoprotein of SARS-CoV-2 [4].

The results of numerous studies indicate that the expression of ACE2 is highest in the small intestines, and high in tissues such as the salivary glands, testicles, kidneys, heart, thyroid, and adipose tissues. On the other hand, ACE2 expression is low in tissues such as the spleen, brain, muscles, pituitary, and skin [5]. However, dermatologists have recently raised concerns about the potential for the virus to infect the skin due to the increasing observations of cutaneous manifestations in patients infected with SARS-CoV-2 [6,7]. The ACE2 receptor of SARS-CoV-2 in the skin has been found to be expressed mainly on keratinocytes in the stratum basale, the stratum spinosum, and the stratum granulosum of the epidermis [7]. These findings provide a novel insight into a potential transmission route through the skin [8]. Although the fomite-mediated transmission of severe acute respiratory syndrome plays a negligible role when working alone, it has a much stronger effect when working in combination with airborne transmission [9]. Presently, there is no clinical evidence of skin-to-skin infection [10]. Nevertheless, the high expression of *ACE2* on keratinocytes in human skin indicates that percutaneous transmission might be a potential route for SARS-CoV-2 infection, especially in cases of conditions causing skin barrier dysfunction [8]. Epidermal barrier integrity could be influenced by various factors involved in epidermal cell differentiation and proliferation, cell–cell adhesion, and skin lipids [11]. The disruption of epidermal homeostasis is associated closely with the deterioration of skin health and the pathophysiology of various skin diseases [12]. Moreover, even psychological stress increases the level of cutaneous glucocorticoids and eventually impairs barrier function [13]. Additionally, skin barrier damage and irritation can also occur due to extensive use of personal protective equipment (e.g., masks, protective suits, and a few others) and predominately alcohol-based sanitizers [14], which were recommended for use during the COVID-19 pandemic. All these cases of the skin barrier weakening, which may be caused by many more factors than those mentioned above, confirm the high demand for agents that protect the skin against SARS-CoV-2 infection.

The World Health Organization, in its guidelines from 19 March 2020, emphasized that during an epidemiological threat, it is of fundamental importance to ensure sanitary and hygienic conditions [15]. One of the most important preventive measures against SARS-CoV-2 coronavirus infection is appropriate hygiene, especially as the main transmission routes are droplets and contact [16]. Therefore, there is a need to find antimicrobial agents that can be used as impregnates for protective clothing or surface and skin disinfectants that are both effective and safe for the skin, which can be exposed to the virus and become a site of infection.

Silver nanoparticles (Ag), diamond nanoparticles, (D), and graphene oxide (GO) are some of the agents that can be used in the maintenance of personal and environmental hygiene and, in limited, non-toxic concentrations, have been proven to have antibacterial, antifungal, and antiviral effects. The mentioned nanomaterials could be a part of new agents/systems for preventing SARS-CoV-2 infections and/or virus transmission etc. So far, studies concerning interactions between GO, Ag, and D with SARS-CoV-2 are scarce.

In particular, the characteristics of Ag nanoparticles make them suitable candidates for applications in the biomedical, food, and environmental industries [17]. Ag nanoparticles induce numerous oxidative and non-oxidative processes including adhesion to surfaces, electrostatic interactions with membranes, cell destruction, inappropriate functioning of organelles, and interaction with proteins and nucleic acids. Ag nanoparticles are employed as antifungal, antibacterial, and antiviral drugs [18,19] for wound dressings, long-term burn care products [20], medical device coatings, medical textiles, orthopaedics, contraceptive devices, cosmetic clothing, lotions for both treatment and supplementary drug and/or nutrient delivery, paints, sunscreens, et cetera [21,22,23]. However, the broad utilization of Ag nanostructures has revealed shortcomings such as instability, binding with major blood proteins, damage to proteins, nucleic acids, and membranes, and immunosuppression of most cytokines [24].

D, thanks to its superior mechanical, tribological, electrical, chemical, and biological qualities, is a choice material for investigating safe antibacterial films, coatings, and particles. Several parameters must be taken into account to understand the D and D-related antibacterial mechanisms of action and antibacterial efficiency. Grain size is important, since only D films made of microcrystalline D display antibacterial properties. The surface functionalisation of films and nanodiamonds is important as it influences the antibacterial properties of these materials. Additional functionalisation or implantation with silver, copper, fluorine, anatase, and zinc oxides is important to create satisfactory antibacterial properties [25].

GO, due to its contact-based antimicrobial activity, is a promising candidate for the creation of materials with antimicrobial properties. Thus, GO can allow for the improvement of textiles used in personal protective equipment [26]. Moreover, the antibacterial activity of reduced GO/Ag nanocomposites has been proven for multidrug-resistant bacterial strains, whereas its cytotoxicity toward different mammalian cells is low [27,28]. Additionally, the synergistic effect of Ag and GO as a complex can activate the epidermis renewal process, making the complex suitable for use in skin contact. Moreover, the complex has a positive effect on the micro- and ultra-structure of a 3D skin model [29]. It did not affect the uniformity of the corneal layer, the sample remained the most compact and was characterized by homogeneous morphology of a corneal layer, and there were also no pathological changes in the ultrastructure.

Therefore, considering their anti-SARS-CoV-2 applications, these nanomaterials and their complexes should be examined for their interaction with ACE2 to determine whether they influence the binding of the virus. Additionally, in the event that they block ACE2/SARS-CoV-2 binding, their lowest effective and non-toxic concentrations should be determined.

## 2. Results

### 2.1. Characteristics of Nanomaterials

**Figure 1:**
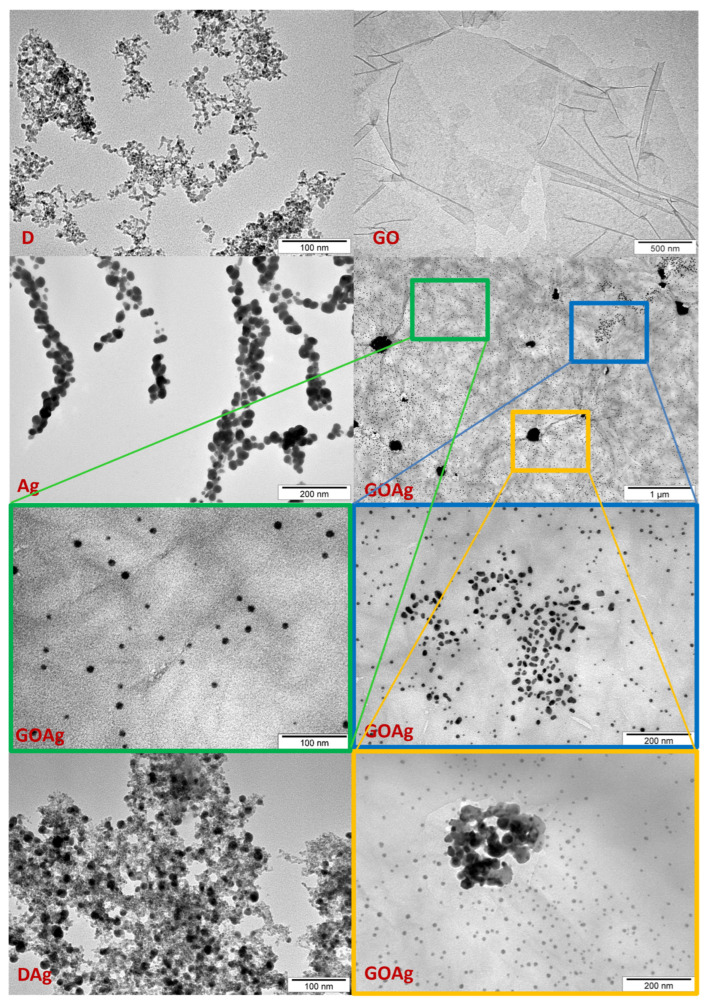
TEM visualization of nanomaterials and their complexes. D—diamond nanoparticles, scale bar reflects 100 nm; GO—graphene oxide, scale bar reflects 500 nm; DAg—nanocomplex of diamond and silver nanoparticles, scale bar reflects 100 nm; GOAg—nanocomplex of graphene oxide and silver nanoparticles, scale bar reflects 1 µm; Ag—silver nanoparticles, scale bar reflects 200 nm. GOAg in the blue and yellow frame—scale bars reflect 200 nm; GOAg in the green frame—scale bar reflects 100 nm.

As depicted in Figure 1, the nanomaterials greatly differed in terms of morphology and dimensions. The organization of the nanomaterials changed depending on whether they were used individually or in mixtures. When Ag was mixed with D, it showed an even distribution, whereas it had a tendency to agglomerate when used alone. When Ag was complexed with GO, Ag was distributed as groups on the surface of GO. The larger the Ag dimensions, the stronger the tendency to aggregate. Therefore, the largest nanoparticles created agglomerations on the GO lattice. The medium-sized Ag dimensions formed groups in which the nanoparticles were close, but not in contact with each other. The smallest Ag nanoparticles were distributed most evenly.

### 2.2. ACE2 Inhibition by Nanomaterials

**Figure 2:**
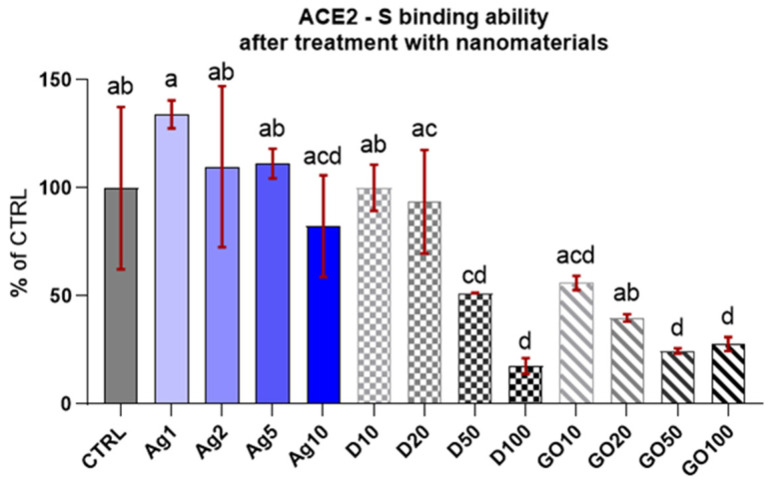
ACE2-S binding in the presence of the specified nanomaterials. Results are presented as % of control (means ± SD, n = 6). *p* ≤ 0.05 was considered a statistically significant difference. Letters a–d above the treatment bars—the same letters indicate no significant differences between treatments, while different letters above treatment bars indicate the presence of statistically significant differences between specific groups.

Initially, a competitive test was conducted to evaluate the capability of the chosen concentrations of individual nanomaterials to block the ACE2 receptor (or bind to the S–RBD protein complex). The concentrations were selected on the basis of the previous research included in the articles cited in the introduction [17,18,19,20,21,22,23,24,25,26,27,28,29], but also based on the unpublished experimental results of our research team. The results (Figure 2) reveal that only high dosages of carbon nanomatherials (D50-100, GO50-100) have ability to block ACE2 and in the case of D, this action is dose-dependent—the higher the concentration, the higher the inhibition against ACE2-S binding. However, the lower concentrations of D and GO demonstrated a similar ability to block ACE2-S binding as the control and Ag (no inhibition).

**Figure 3:**
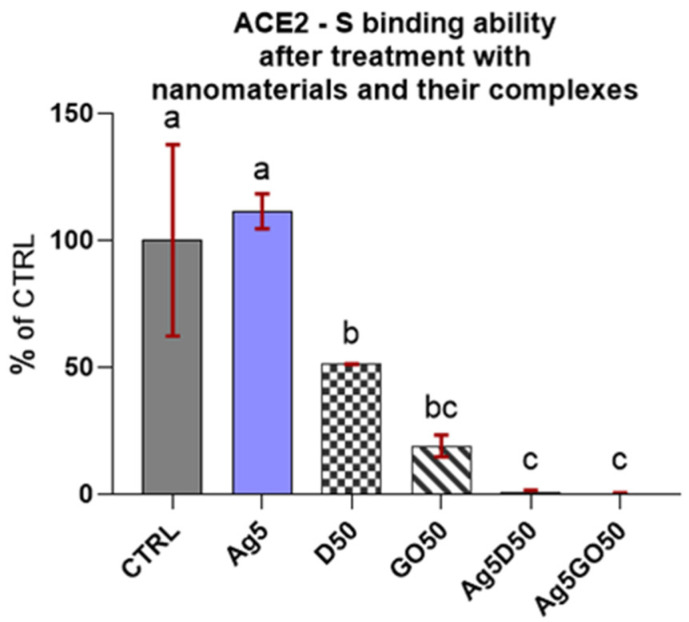
ACE2-S binding in the presence of specified nanomaterials. Results are presented as % of control (means ± SD, n = 6). *p* ≤ 0.05 was considered a statistically significant difference. Letters a–c above the treatment bars—the same letters indicate no significant differences between treatments, while different letters above treatment bars indicate the presence of statistically significant differences between specific groups. Nanomaterial concentrations refer to micrograms per millilitre.

The results of the competitive tests for blocking of the ACE2 receptor by individual nanomaterials in comparison to their complexes and the control group (Figure 3) indicated that Ag complexes with carbon nanomaterials preserved ACE2-S binding. This effect was as strong as in the blank (subtracted from all the reads), where the S protein was not added to the well, which means these nanocomplexes have the maximum inhibitory capacity. D and GO nanomaterials also exhibited robust inhibition and Ag alone had no influence on ACE2-S binding.

### 2.3. Viability and Morphology of Cell Line Cultures Treated With Single Nanomaterials at Different Concentrations

**Figure 4:**
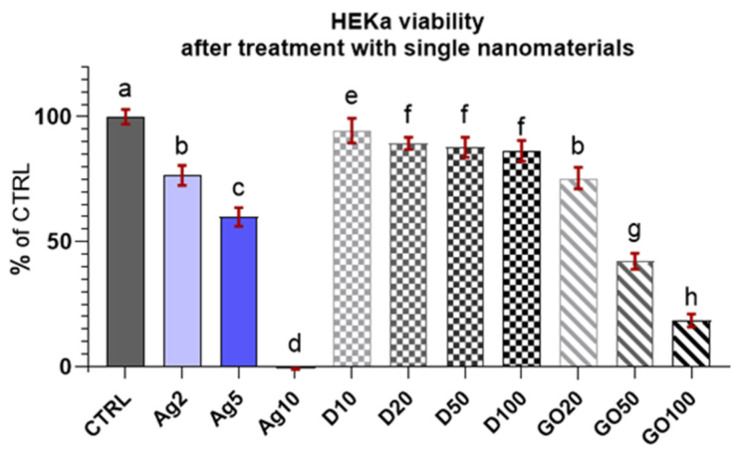
Viability of HEKa cells treated with different concentrations of carbon nanostructures and silver nanoparticles. Results are presented as % of control (means ± SD, n = 6). *p* ≤ 0.05 was considered a statistically significant difference. Letters a–h above the treatment bars—the same letters indicate no significant differences between treatments, while different letters above treatment bars indicate the presence of statistically significant differences between specific groups. Nanomaterial concentrations refer to micrograms per millilitre.

The results of the viability test for the HEKa cells (Figure 4) indicated that the cells were sensitive to nanomaterials and even low concentrations lead to cell mortality, especially in the case of Ag, where a concentration of 10 µg/mL killed all the cells. The results are presented as a % of the control (means ± SD, n = 6). A decrease in HEKa cell viability was also observed with the application of GO. The reactions of the HEKa cells with the D concentrations was slightly different. D also lowered the viability of cells; however, its action was not as drastic as that of the other nanomaterials, and the viability of the cells was >80%.

**Figure 5:**
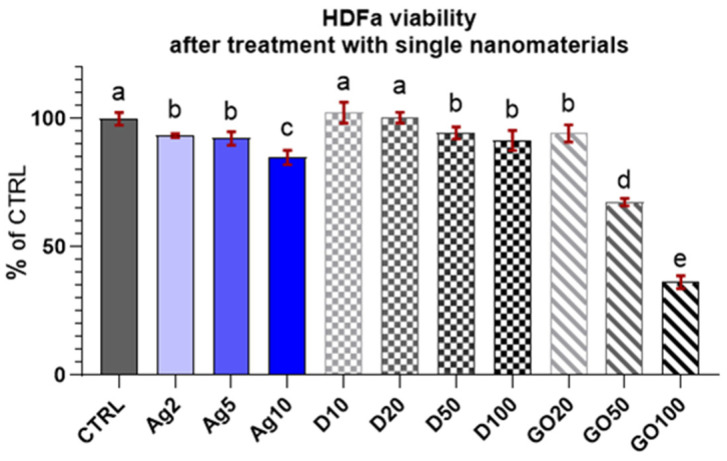
Viability of HDFa treated with different concentrations of carbon nanostructures and silver nanoparticles. Results are presented as % of control (means ± SD, n = 6). *p* ≤ 0.05 was considered a statistically significant difference. Letters a–e above the treatment bars—the same letters indicate no significant differences between treatments, while different letters above treatment bars indicate the presence of statistically significant differences between specific groups. Nanomaterial concentrations refer to micrograms per millilitre.

The results of the viability test for the HDFa cells (Figure 5) demonstrated that they were less sensitive to nanomaterial concentrations compared to the HEKa cells. Surprisingly, GO was the most toxic nanomaterial at high concentrations for these cells. Ag and D had a similar influence on cell viability, slightly lowering the level. The results looked promising, especially in the case of D, where none of the concentrations caused a greater toxic effect. For Ag, the viability still remained at high level (>80%) in comparison to the HEKa cells.

A multi-factor ANOVA showed that the viability of cells significantly differed between the HDFa and HEKa cell lines; however, it could not detect significant differences between the lines among particular groups as the viability of cells in control groups was specific for each line. Therefore, to compare the viability of both cell lines treated with particular nanomaterials, viability coefficients with expanded standard uncertainty were calculated, referencing individual results to the control (Table 1).

By comparing the HEKa and HDFa viability coefficients after treatment with a single nanomaterial, it can be assumed with 95% certainty that for both cell lines, the concentrations of D10–100 were not harmful and did not cause increased mortality. In contrast, for GO, high concentrations of GO20–100 caused a marked decrease in the viability of the HEKa cells and, to a limited extent, of HDFa cells following a dose-dependent pattern in both cases. Regarding Ag, differences were visible between the two cell lines. The HEKa cells were highly susceptible to this nanomaterial, while a lowering of cell viability was dose-dependent. The HDFa cells were not influenced by low concentrations of pure Ag (Ag2 and Ag5). At this research stage, the main factor influencing the toxicity of the nanocomplex could not be determined, but GO and Ag are the probable candidates.

**Figure 6:**
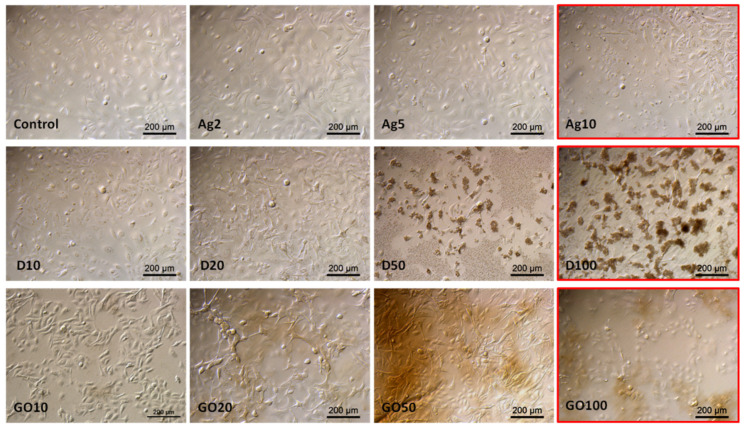
HEKa morphology in a culture treated with different concentrations of carbon nanostructures and silver nanoparticles. The red frame indicates major morphological changes in cells treated with the highest concentration of nanomaterials. Ag—10 µg/mL, D—100 µg/mL, GO—100 µg/mL. Scale bar 200 µm.

The observations made in the HEKa cell culture confluence confirmed the viability test results. The highest concentrations considerably lowered the confluence of cell layers and, in the case of carbonic nanomaterials, caused their agglomeration, enabling their visibility under the magnification of the optical microscope (Figure 6).

**Figure 7:**
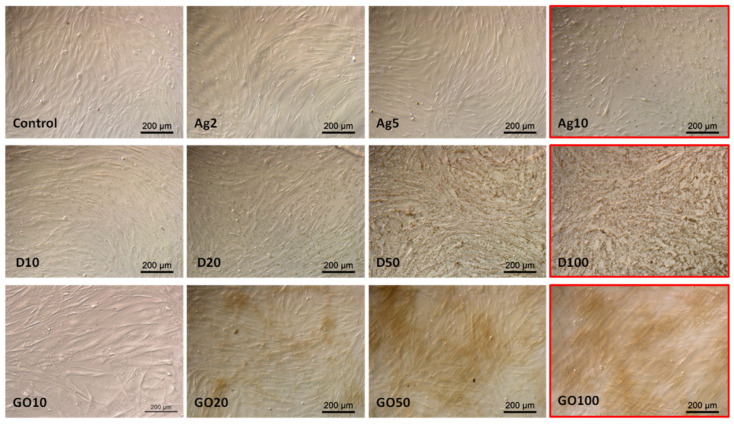
HDFa morphology in a culture treated with different concentrations of carbon nanostructures and silver nanoparticles. The red frame indicates major morphological changes in cells treated with the highest concentration of nanomaterials. Ag—10 µg/mL, D—100 µg/mL, GO—100 µg/mL. Scale bar 200 µm.

Observations of HDFa morphology also confirmed the viability tests results; however, even high concentrations did not significantly change the cell morphology (Figure 7). In the case of Ag, high concentrations caused cell dehydration. D also accumulated on the surface of the HDFa cells, but its absorption by the cells could not be observed at this magnification.

The results of the viability tests, viability coefficients, and morphological observations of HEKa and HDFa cells treated with different concentrations of single nanomaterials were taken into account. For the next experiments with the nanomaterial complexes, only low concentrations were used. The highest concentrations, marked in Figure 6 and Figure 7 with a red frame, were excluded from further investigations.

### 2.4. Viability and Morphology of Cell Line Cultures Treated with Complexes of Different Nanomaterials

**Figure 8:**
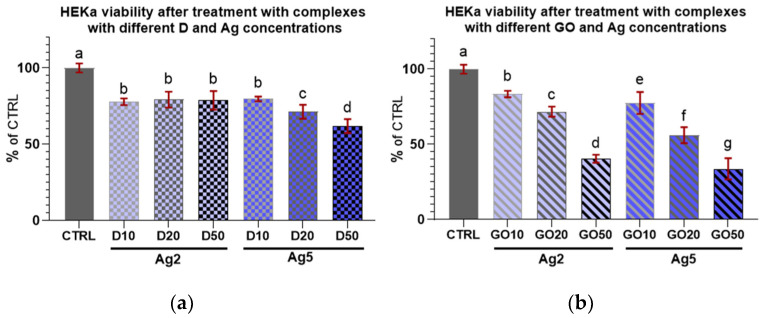
Viability of HEKa cells treated with (**a**) diamond and (**b**) graphene oxide complexes with silver nanoparticles of different concentrations. Results are presented as % of control (means ± SD, n = 6). *p* ≤ 0.05 was considered a statistically significant difference. Letters a–g above the treatment bars—the same letters indicate no significant differences between treatments, while different letters above treatment bars indicate the presence of statistically significant differences between specific groups. Nanomaterial concentrations refer to micrograms per millilitre.

The results for HEKa cell viability after treatment with D and GO complexes with Ag can be seen in Figure 8. It can be generally concluded that all nanocomplexes decreased cell viability.

Regarding the complexes with D (Figure 8a), an initial increase in nanomaterial concentration in the complex did not induce a decrease in viability among the treated cells; furthermore, there were no differences in viability in the cultures treated with all D complexes with Ag at a concentration of 2 µg/mL.

In the case of complexes with GO (Figure 8b), Ag at both concentrations decreased HEKa cell viability in a dose-dependent manner. It was also noted that every increase in the concentration of any component in the nanocomplex caused a significant decrease in cell viability.

**Figure 9:**
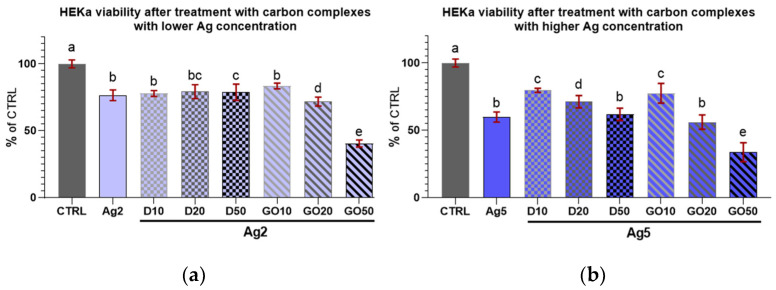
Viability of HEKa cells treated with diamond and graphene oxide complexes with silver nanoparticles at a concentration of (**a**) 2 µg/mL and (**b**) 5 µg/mL. Results are presented as % of control (means ± SD, n = 6). *p* ≤ 0.05 was considered a statistically significant difference. Letters a–e above the treatment bars—the same letters indicate no significant differences between the treatments, while different letters above treatment bars indicate the presence of statistically significant differences between specific groups. Nanomaterial concentrations refer to micrograms per millilitre.

In the case of D complexes with a low concentration of Ag (2 µg/mL) and Ag treatment alone, the viability of the keratinocytes was slightly lowered compared to the control (Figure 9a) and remained at the same level independent of D concentration. For the GO complexes with Ag2, the viability decline was dose-dependent; however, the lowest concentration of GO (10 µg/mL) seemed to offer protection against Ag2 toxicity to cells.

Regarding the high concentration of Ag (5 µg/mL) in the complexes with D, increasing the D concentration caused a systematic lowering of HEKa cell viability (Figure 9b). However, the viability of cells treated with pure Ag at a concentration of 5 µg/mL was at the same level as that of the group treated with the D50Ag5 nanocomplex, and a low concentration of D was protective against Ag5 toxicity.

**Figure 10:**
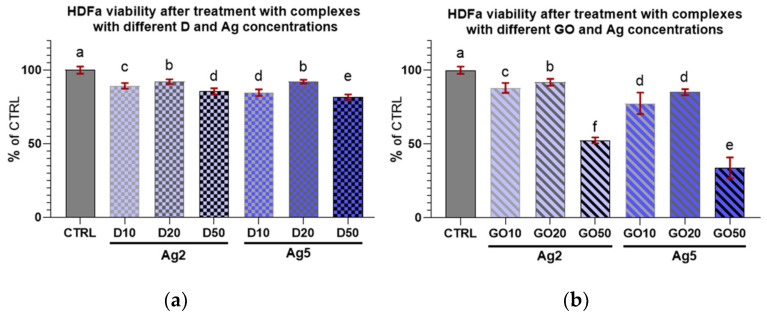
Viability of HDFa treated with (**a**) diamond and (**b**) graphene oxide complexes with silver nanoparticles of different concentrations. Results are presented as % of control (means ± SD, n = 6). *p* ≤ 0.05 was considered a statistically significant difference. Letters a–f above the treatment bars—the same letters indicate no significant differences between treatments, while different letters above treatment bars indicate the presence of statistically significant differences between specific groups. Nanomaterial concentrations refer to micrograms per millilitre.

**Figure 11:**
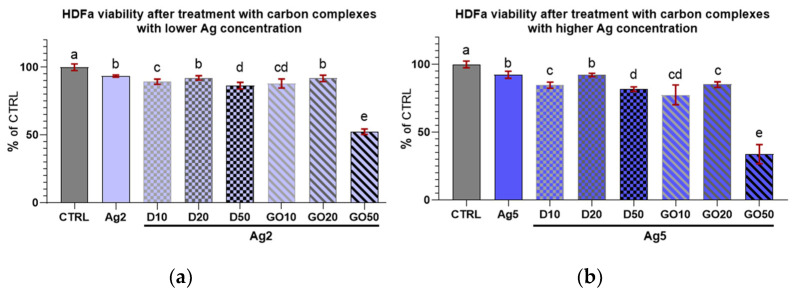
Viability of HDFa treated with diamond and graphene oxide complexes with silver nanoparticles at concentrations of (**a**) 2 µg/mL and (**b**) 5 µg/mL. Results are presented as % of control (means ± SD, n = 6). *p* ≤ 0.05 was considered a statistically significant difference. Letters a–e above the treatment bars—different letters indicate significant differences between treatments, while different letters above treatment bars indicate the presence of statistically significant differences between specific groups. Nanomaterial concentrations refer to micrograms per millilitre.

Concerning HDFa cell viability after treatment with the nanocomplexes (Figure 10 and Figure 11), it can be seen that every treatment lowered cell viability. However, these cells seemed to be less sensitive than the keratinocytes to the influence of the nanocomplexes. Only high concentrations of nanomaterials, especially GO, caused a marked decrease in the viability of these cells.

When comparing the viability coefficients of the HEKa and HDFa cells after treatment with nanocomplexes, it can be inferred that for both cell lines, most concentrations, regardless of the nanocomplex composition, caused a decrease in cell viability. Regarding the Ag2 complexes with D, for all concentrations, the viability of cells remained at the same level for each cell line. For the Ag2 complexes with GO, increasing concentrations of GO increased the mortality in both cell lines. For the Ag2GO50 complex, the viability of HEKa and HDFa cells was lower than in the group treated with pure Ag2. For the Ag5 complexes, the results for the HEKa cells were surprising. The addition of the lowest concentration of D nanoparticles (D10) seemed to diminish the mortality caused by pure Ag5. However, the highest D concentration (D50) and all GO (GO50) concentrations in complexes with Ag5 decreased cell viability in both cell lines to below the level in the Ag5 group. In conclusion, for both the HEKa and HDFa cell lines, cell viability was influenced mostly by Ag and carbon nanomaterials; especially by GO at high concentrations, which modulated the effects of Ag.

As with singular nanomaterials, to compare the influence of nanocomplex treatments on the viability of particular cell lines, viability coefficients were calculated by referencing the individual group results to the control group (Table 2).

Table 2 also depicts the differences in the viability coefficients between the two cell lines. The difference between cell lines in the same experimental group is significant when the values of coefficients ± uncertainty do not overlap. In the experimental groups, in most cases of high concentrations of D and GO in complexes with Ag5, the viability of the HEKa cells was strongly influenced by the nanomaterials added to the culture medium. However, for the most of concentrations of D and GO in the complexes with Ag2, the differences between the HEKa and HDFa cells were not pronounced. Although differences existed in the Ag2D10 and Ag2GO50 groups, in both cases, the HEKa cells were more susceptible to treatment than the HDFa cells.

**Figure 12:**
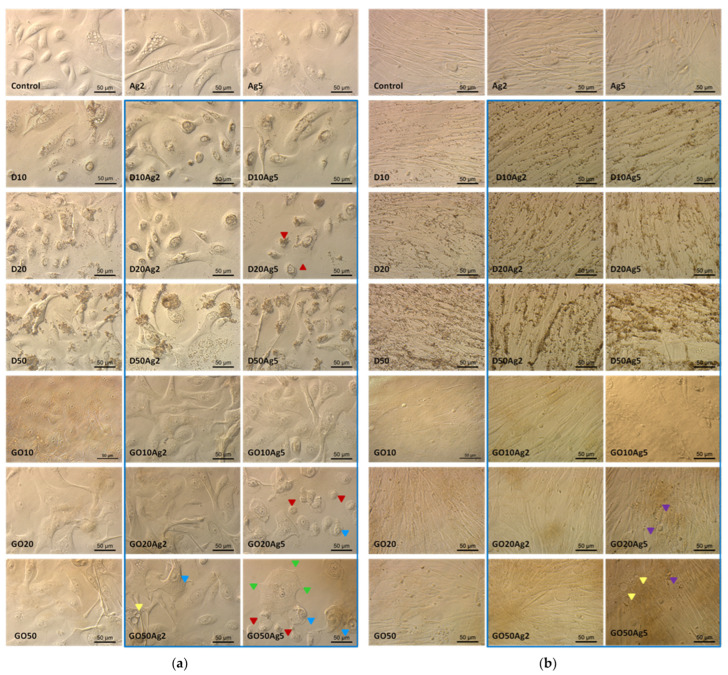
HEKa (**a**) and HDFa (**b**) cell morphology in a culture treated with carbon nanostructures, silver nanoparticles, and their complexes (blue frame). Red arrowheads—shrunken cells; yellow arrowheads—vacuolized cytoplasm; green arrowheads—shortened filopodia; blue arrowheads—nuclear chromatin accumulated near the nuclear membrane; purple arrowheads—apoptotic bodies visible as protrusions from the plasma membrane.

Regarding HEKa and HDFa morphology after treatment with single nanomaterials and nanocomplexes (Figure 12a,b), it can be seen that only the lowest concentrations of these materials did not cause visible morphological changes in the cells. However, the question arose as to whether these concentrations would be sufficient to block ACE2 on the cell surface.

Another interesting difference between HEKa and HDFa cell cultures concerns the distribution of D and AgD agglomerates on the surface of cell membranes. Keratinocytes (Figure 12a) seemed to internalize these nanomaterials, and their agglomeration took place in the vesicles inside the cell, whereas in the case of fibroblasts (Figure 12b), agglomerates accumulated outside the cells in the grooves between individual cells.

Figure 12 also confirms the results of the viability coefficient comparisons between the HEKa and HDFa cell lines. Morphological changes were specific for treated cells and did not occur in control group. They were more visible for keratinocytes, which are more susceptible to treatments with nanomaterials. It can be seen that keratinocyte morphology in the groups treated with Ag5 and its complexes distinctly displayed characteristics of reduced viability and cell death, namely, reduced confluence of the culture, cell dehydration features, numerous vacuoles inside the cell cytoplasm, or reduction in the number and length of the filopodia. Cultures treated with Ag5 in complexes with high concentrations of GO cells displayed morphological characteristics of early apoptosis. The apoptotic cells separated from the others. As a result of the loss of intracellular water and electrolytes, the cells shrunk and showed changes in the shape, size, and density of the cytoplasm. Changes in the nuclear chromatin were also visible, with accumulation occurring near the nuclear membrane [30]. Another characteristic feature of apoptosis was the formation of apoptotic bodies which were visible in some places as protrusions from the plasma membrane, commonly referred to as ‘blebs’ [30], especially in fibroblasts treated with Ag5GO20-50.

## 3. Discussion

### 3.1. Characteristics of Nanomaterials

The physicochemical characteristics of the nanomaterials used in this experiment are crucial for their interaction with living cells and organisms. In our work, the sonication method of working solutions, compared to the former method used by our research team [31], made a difference to the behaviour of the nanomaterials and complexes in the solutions. As seen in the TEM images of the AgGO complexes and Ag nanoparticles, the nanomaterials were distributed differently in relation to each other [31] compared to their distribution after a shorter but more intense sonication cycle. Even a very similar method by Pruchniewski [32] and Zielińska-Górska applying the same sonic instrument with different time conditions [33] caused differences in the nanocomposite structures.

During the fabrication of the GO films, the mechanical properties, such as the strength and size of the final GO films, were dependent on sonication time. Prolonged sonication gradually deteriorates these mechanical properties [34], leading to a reduction in size and weakening of sheets. For metallic nanoparticles, probe sonication has been the preferred method for dispersing non-inert, non-functionalized metal nanoparticles (Cu, Mn, and Al). However, after sonication, strong van der Waals forces of the metal nanoparticles resulted in extensive agglomeration [35]. Therefore, it is possible that strong sonication not only improves the dispersion of nanomaterials in water solutions but can also influence the quality, quantity, and distribution of functional groups on the surface of nanomaterials, which would greatly change their key properties. Nevertheless, in our opinion, the agglomeration of nanomaterials is not the same as their solidification, and the resulting porosity of agglomerates is an advantageous feature in applications related to the binding (blocking or transporting) of different particles. From our point of view, sedimentation, in turn, would be a useful phenomenon because it could fasten the contact of nanostructures with cells in the culture.

The size of the nanomaterials and nanocomplexes is an important feature. Taking into account the expected surface applications of resulting formulations, it would be safer if the nanomaterials were not internalized by cells. When comparing our TEM images of nanomaterials with those specified by the manufacturer’s dimensions, it can be seen that their size ranges are broader than declared. In the cases of the D and Ag nanoparticles, larger nanoparticles (D above 6 nm and Ag above 30 nm) are visible on the micrographs, whereas in the case of GO nanoflakes smaller than 5 µm are present. The characteristics of most of the nanomaterials examined previously [32,36] proved that the Ag nanoparticles, at least, are larger than declared.

### 3.2. ACE2 Inhibition by Nanomaterials

The ACE2-S binding competitive test showed that in the case of D and GO, the concentration of the nanomaterial caused a dose-dependent blocking effect (Figure 2). A high concentration results in a lowered likelihood of ACE2 binding with S-RBD, indicating that in high concentrations, carbon nanomaterials had the ability to directly block ACE2 or bind to S-RBD, thereby counteracting the ACE2–S binding. In both cases, the application of these nanomaterials might be effective in protecting the skin against SARS-CoV-2. These results were used to determine the individual nanomaterial concentrations suitable for the preparation of nanocomplexes.

The concentrations used in the experiment were chosen on the basis of earlier research on their toxicity levels, which vary depending on the cell line, in vitro model, exposure time, and dosage [37,38,39,40,41,42]. However, we anticipated that combining different nanomaterials at high concentrations into complexes could cause unexpected merged toxicity resulting from physical contact and chemical interactions with cell membranes, intracellular organelles, and biological compounds. Therefore, we avoided mixing the highest concentrations of nanomaterials. The results confirmed our predictions—the blocking capacity of the resulting nanocomplexes was stronger compared to that of individual nanomaterials. It remains to be seen how particular concentrations of nanomaterials and their complexes influence the viability of human skin cell lines (Figure 3).

### 3.3. Viability and Morphology of Cell Line Cultures Treated with Single Nanomaterials of Different Concentrations

In our experiments, the inhibition concentration of 50% (IC_50_) for 24 h cultures in vitro was achieved or exceeded when the calculated viability coefficient values were equal to or less than 0.5. The results showed that for both of the cell lines examined, all concentrations of D did not achieve the IC_50_ level. Moreover, upon analysing the calculated expanded standard uncertainty of the viability measurements, none of the viability coefficients calculated for all groups treated with D differed from the viability coefficient of the control group with 95% certainty. Differences between the HEKa and HDFa cells appeared for GO. For this nanomaterial, the decrease in viability was highly dose-dependent; for HDFa, the highest concentration exceeded IC_50_, and for HEKa cells, only the lowest concentration was below this value. Moreover, all the viability coefficients differed from the viability coefficient of the control group. The most pronounced differences between cell lines were observed after Ag treatment, where for HDFa cells, all of the concentrations utilized were far lower than IC_50_, and the viability coefficient was lower than the coefficient of the control group but only for the highest concentration. For the HEKa cells, the highest concentration of Ag was completely lethal and the concentration of 5 µg/mL, although below IC_50_, greatly decreased cell viability (Table 1 and Figure 4 and Figure 5).

Naturally, the question arises as to whether the results of in vitro studies can be extrapolated to in vivo conditions. Extensive in vitro studies, with cultured human skin fibroblasts (WS1, CRL-1502; Detroit551, ATCC-CCL-110; ATCC, Manassas, VA, USA), performed to test the basal cytotoxicity theory revealed that the experimental half maximal inhibitory concentration values for cell cultures are as accurate predictors of human toxicity as the equivalent toxic blood concentrations derived from rodent LD_50_s [43].

Regarding the toxicity and proliferative potential of GO, many conflicting findings have been reported. For example, multi-layer GO shows a proliferative activity similar to that of epidermal growth factor after 96 h, in contrast to the antiproliferative effect of few-layer GO reported for human keratinocyte HaCaT cells. Moreover, multi-layer GO has been shown to be more toxic than the previously reported few-layer GO for the same cell line [44]. When GO toxicity was assessed based on oxidation level, elemental composition, and size, the results indicated that elemental composition and size have an impact on GO toxicity, while oxidation level has no significant effect. GO with significantly high carbon–carbon and carboxyl groups showed a high toxicity level. The toxicity levels of sonicated GOs tend to increase as their sizes decrease [45].

Nanodiamond has gained increasing attention owing to its biocompatibility. Biocompatibility studies on nanodiamonds produced through the detonation technique (used in our experiments) have been explored extensively. The 2D and 3D toxicity profiles of nanodiamonds created through other methods, for example, laser-assisted techniques, differ. Cytotoxic effects, however, were not observed in the case of the 3D model for any nanodiamonds produced through any methods [46]. Similar conclusions were drawn by researchers in our team and co-workers. Their results indicated that nanodiamonds had no toxic effect at a concentration of 25 µg/mL tested on a reconstituted human epidermis (EpiDerm^TM^, MatTek, Bratislava, Slovakia). They also did not adversely affect the tissue structure and did not lead to a simultaneous increase in protein and mRNA expression of the analysed cytokines, which confirms the safety and biocompatibility of nanodiamonds for application in skincare [47]. These observations were also confirmed in our experiments (Table 1) which showed that for HEKa and HDFa cells, even a high concentration of 100 µg/mL did not cause a reduction in cell viability. This allows us to expect potential therapeutic effects from the external application of nanodiamonds and their Ag complexes to the skin.

During the coronavirus pandemic, highly purified detonation nanodiamonds (DNDs) successfully inactivated various key cytokines in plasma from cytokine release syndrome (CRS) patients by adsorbing inflammatory cytokines in patients with pneumonia and septic shock. Also, the multiple intravenous injection of the DND samples in a CLP mouse model improved survival, while suppressing blood vessel disruption and pulmonary inflammation [48]. On the other hand, it was also observed that for engineered nanodiamonds, even non-cytotoxic concentrations increased nitric oxide and reactive oxygen species production, resulting in sustained oxidative/nitrosative stress and causing imbalances in energy metabolism and mitochondrial malfunctioning [49]. The other teams reported cell-specific and material-specific toxicity for the different types of diamond nanoparticles tested. These results, in turn, demonstrated the role of purification and modification methods on the properties of DND particles and their cytotoxicity, as well as the importance of cell types used for the evaluation of the nanomaterials [50,51]. Consequently, there is still an urgent need to examine the impact of all new nanomaterials and nanocomplexes on cell/tissue metabolism in the long term, while considering the chemical and physical changes of the factors studied.

Finally, the differences between the HEKa and HDFa cell cultures also concerned the distribution of D and AgD agglomerates (Figure 12a,b). Keratinocytes seemed to internalize these nanomaterials, and their agglomeration took place in the vesicles inside the cell, in a perinuclear area, whereas in the case of fibroblasts, agglomerates accumulated outside the cells. This phenomenon was observed for many different healthy and cancerous animal and human cell lines, among which a number of healthy epithelial cell lines of different types internalised the diamond nanoparticles. It has been observed that diamond particles with sharp edges leave the endosomes. Rounded diamonds are also ingested by some cells. However, they remain in the endosomes and are finally excreted again [52,53,54,55,56,57]. The second mode seems to be consistent with our observations in keratinocytes (Figure 12a) where, especially after applications of D or DAg in high concentrations, agglomerates remain attached to the cell surface.

### 3.4. Viability and Morphology of Cell Line Cultures Treated with Different Nanomaterial Complexes

Research by Samberg et al. on human epidermal keratinocytes revealed that carbon-coated Ag nanoparticles caused no significant decrease in the viability of cells and were localized on the surface and in the upper stratum corneum layers of the skin [38]. The same results could probably be expected from our Ag complexes with 10 ppm concentrations of D and GO because they also seemed to protect keratinocytes from the toxicity of Ag5 and the decrease in cell viability in these cases was marginal (Figure 11b).

Unfortunately, regarding further applications, our findings revealed a synergistic toxicity effect of the GOAg nanocomposite at high concentrations of both components (Table 2 and Table 3) for both cell lines. Similar findings were reported by de Luna et al., who found that the GOAg nanocomposite was more toxic than pristine GO and pristine Ag nanoparticles to tumoural J774 macrophages. TEM analysis showed that GOAg was internalized by macrophages, but the images also revealed the degradation of nanocomposites inside the cells [58], which seems to be promising. Therefore, according to our results, the best parameters related to cell survival (cell viability and proper morphology) were revealed by cells after the treatment with AgD nanocomplexes, which had lower cytotoxicity for both cell lines but especially for HDFa cells, rather than Ag nanocomplexes with GO (Table 2). Moreover, we assumed that for skin fibroblasts, the way the cell contacts the nanocomplex is safe considering the possible aforementioned intracellular disintegration of Ag complexes with carbon nanomaterials [44,58].

Contrary to our expectations, there is only limited data in the literature concerning the research/applications of DAg nanocomplexes. Existing studies have focused on nanocomposites containing diamond-like carbon thin films doped with Ag nanoparticles. These nanocomposites have been utilized in medical implants with strong potency against microbial infections [59] and also, more importantly to us, in preclinical studies of patches for skin wound treatment [60]. Research using laboratory animals demonstrated that the patch prototype was able to kill all MRSA bacteria strains in the wound’s bed after 72 h of treatment [61]. This finding gives hope for the multifaceted potential of our DAg nanocomplexes. When applied to damaged skin, especially in the context of the increased incidence of SARS-CoV-2, these nanocomplexes could protect damaged vessels in which endothelium ACE2 is expressed [61] against viral infection and safeguard the wound against microbial infection.

### 3.5. General Remarks

Cell lines grown in vitro are devoid of the natural protective mechanisms that are present in living tissue. Consequently, the slight reduction in cell line viability observed in our studies does not necessarily have to be a result of the toxicity of the tested nanomaterials. In our opinion, only obvious results of reduced cell viability close to IC_50_ may constitute a reason to refrain from using specific concentrations in further studies. Generally, our results show that HEKa cells are more sensitive to all Ag treatments and higher dosages of GO (20 and 50 µg/mL) compared to HDFa cells (Table 1). A similar situation occurred in the case of treatments with nanocomplexes, but we could see that nanocomplexes of highly concentrated carbons (20 and 50 µg/mL) with Ag5 particularly deepened the differences between the cell lines examined (Table 2). While these results could be interpreted as disturbing, mammalian skin is fortunately equipped with a highly dynamic stratified epithelium. In the epidermis, keratinocytes comprise four layers and three other cell types, besides keratinocytes, play various roles [62].

The dermis is a differentiated connective tissue which provides flexible and resistant support to the epidermis and allows the diffusion of nutrients from the bloodstream to the ectodermal cells [63]. Dermal fibroblasts are a dynamic and diverse population of cells, the functions of which in the skin remain unknown in many respects [64]. Generally, fibroblasts are diverse mesenchymal cells that play a role in tissue homeostasis and disease by producing a complex extracellular matrix and creating signalling niches through biophysical and biochemical cues [65]. Normal adult human skin contains at least three distinct subpopulations of fibroblasts, occupying unique dermis niches [64]. Papillary-, reticular-, and hair follicle-associated fibroblasts differ not only topographically but also functionally [66]. Fibroblasts from each of these niches exhibit distinctive differences when cultured separately. Fibroblasts play an important role in cutaneous wound repair and an increasing role in the bioengineering of the skin [64]. Further, these differences seem to be dictated by the local biological and physical microenvironment in which the fibroblasts reside, resulting in ‘positional identity or memory’ [67]. The association of fibroblast plasticity and heterogeneity with wound healing suggests that a switch in fibroblast phenotype may affect wound healing [68]. Basally located keratinocytes which display stem cell properties, including lifelong proliferative potential and the ability to undergo diverse differentiation trajectories, play a role in the maintenance and regeneration of the epithelium [69]. The stratum corneum (SC) is the first line of defence against physical and chemical attacks. The SC is physically tough and chemically inert and comprises corneocytes, elements which are vital to its barrier function. Corneocytes are differentiated, dead keratinocytes that lack organelles and are composed of a cornified envelope and a keratin-filled interior connected by corneodesmosomes [70,71]. The cornified cells of the SC have a monolayer of lipids, covalently attached to the outer surface of the cornified envelope as the continuous path. Transdermal permeation requires diffusion through this lipid layer [72], which is essential for proper barrier functioning of the skin [72,73]. Of course, there are differences across regions and species in the dermal absorption of compounds and differences in the thickness of the SC substantially contribute to these differences [62]. However, data on the dermal penetration of different types of nanoparticles (with different compositions, dimensions, and shapes) show that the skin is an efficient barrier against nanoparticles, regardless of their properties. While some studies have reported that a small percentage of the applied nanoparticle dose penetrates the skin surface and reaches deep skin layers, a figure of 1% could be used as a worst case dermal penetration scenario [74].

Bearing in mind the results of the toxicity study of the tested nanomaterials on skin cell lines, it is necessary to consider the role played by the individual layers of this important organ. The mammalian epidermis, the outer layer of the skin, is a renewing tissue that forms the outer surface coat of the body. The high impermeability of the SC membrane and its physical strength protects the body against external elements [75]. Additionally, the epidermal water barrier established by the cell envelop, a layer of insoluble proteins on the inner surface of the plasma membrane formed by cross-linking of small proline-rich proteins and large proteins such as cystatin, desmoplakin, and filaggrin, contributes to its strong barrier properties [76,77].

In healthy skin, keratinocytes play a role in maintaining skin homeostasis by actively communicating with fibroblasts. Keratinocytes stimulate fibroblasts through the production of interleukin 1, inducing keratinocyte growth factor (KGF) and metalloproteinases in the fibroblasts. A number of studies have shown that fibroblasts modulate keratinocyte viability, proliferation, and differentiation, with KGF production by fibroblasts being key to these functions [78]. Therefore, despite the relative sensitivity of keratinocytes to the examined nanomaterials, it is important to note that in vivo, keratinocytes are protected by the exfoliating SC and stimulated to proliferation and differentiation by a variety of factors.

SARS-CoV-2 variants show several mutations at the receptor binding domain (RBD) in the spike (S1) glycoprotein, which contribute to immune escape and enhance binding with ACE2. However, genetic variation concerns not only the S1 RBD but also the ACE2 protein in human populations. All these mutations are considered to be the driving forces of viral evolution [79]. This may be perceived as a limitation for our nanocomplexes’ application, but in our opinion the mechanism of blocking is rather physicochemical, whereby specific groups, which are very numerous and quite diverse on the surface of the nanocomplexes, probably bind proteins in a less specific manner, not necessarily by blocking active domain but by changing the protein conformation, for example. That is why ACE2 and S should be perceived rather generally. Following this line of reasoning, our nanocomplexes should act on different variants of the virus and the receptor, and probably on the other types of viral skin infections.

For now, it is too early to recommend any specific way of applying nanocomplexes. Any formulation would be possible, but it first has to be examined in terms of the vehiculum composition and active substance concentration to retain nanocomplex activity and safety. However, in our opinion, even at the stage of suspension it could be incorporated into wound dressings or fabrics for the production of protective clothing.

## 4. Materials and Methods

### 4.1. Materials

#### 4.1.1. Nanomaterials

##### Hydrocolloidal Ag Nanoparticles

Hydrocolloidal Ag (Nano-Tech, Warszawa, Poland) nanoparticles were produced through non-explosive, high-voltage technology (*aXonnite*), using a high-purity metal (99.99%) and demineralized water. *AXonnite* technology is an innovative method of obtaining non-ionic *aXonnite* nanoparticles of precious and semi-precious metals. This technology allows the breaking down of pure metal into particles with sizes ranging from a few to several nanometres in laboratory conditions. Metallic silver Ag4N obtained in the rod form was fragmented by laser ablation into particles which were spherical to oval in shape [80]. The colloids contained Ag at a concentration of 50 µg/mL. The declared size of the nanoparticles ranged from 2 nm to 35 nm. Additionally, the product was characterised previously [36], where the size distribution with the hydrodynamic diameter (109 ± 1.9 nm) was measured with the dynamic light scattering technique (DLS). The ζ-potential of the Ag nanoparticles in ultrapure water (−51 ± 1.4 mV) was determined by the microelectrophoretic method with the Smoluchowski approximation. The morphology of the nanoparticles was evaluated using transmission electron microscopy (TEM).

##### Nanodiamond Powder

Extra-pure detonation nanodiamond powder of the brand PL-D-G02 was obtained from Plasmachem GmbH, Berlin, Germany. The average particle size of the powder was 4–6 nm, the specific surface area was >350 m^2^, the form dry powder, there were traces of non-diamond carbon content, the controlled admixtures % of Fe was <0.05, the sum of other metals was <0.01, the ash content was <0.1%, and the pycnometric density was circa 3.18 g/cm^3^. A more thorough qualitative analysis of PL-D-G02 in comparison to nanodiamonds of other manufacturers was performed by Fourier transform infrared spectroscopy as photoacoustic (FTIR–PAS), diffuse reflectance infrared fourier transform (DRIFT), and attenuated total reflection (ATR) modalities [81]. According to Volkov, D.S. et al. [81], the assignment of characteristic bands showed that only six groups of bands were present in the spectra of all the modalities with appropriate sensitivity: 1760 (C=O stretch, isolated carboxyl groups); 1640–1632 (H–O–H bend, liquid water–the presence of this band is evidence of the water layer at the surface of the nanodiamonds that determines the colloidal solubility of nanodiamonds, this band does not disappear even after five hours of drying); 1400–1370 (non-carboxyl C–O–H in-plane bend and CH2 deformation); 1103 (non-carboxyl C–O stretch); 1060 (in-plane C–H bend, non-aromatic hydrocarbons and carbohydrates), and 940 cm^−1^ (out-of-plane carboxyl C–O–H bend). For FTIR–PAS (IMF, 1.6 kHz), the shapes and positions of the band maxima are reproduced for all of the types of nanodiamonds examined. For ATR–FTIR and DRIFT, the shape of all the test bands and the positions of the maxima are also reproduced, except for the 1750 cm^−1^ band (carboxyl C=O), where a small scatter is observed after vector normalization [81]. The repeatability of the obtained spectra confirms the identity of the nanodiamond we have chosen for the experiments.

##### Water-Dispersed GO

Water-dispersed GO was purchased from NanoPoz Company (Poznan, Poland). The GO was obtained by oxidation of graphite using the modified Hummers method [82] with a 36% oxygen concentration. The GO produced contained carbonyl, carboxyl, hydroxyl, and epoxy groups, confirmed through FTIR analysis. The water-dispersed GO had a concentration of 4000 mg/L and contained flakes with typical diameters of 5–30 µm and a single layer thickness of 0.8–1.2 nm. The pH of the dispersion was neutral. This product was additionally characterised previously by our team: Strojny-Cieślak [36], Zielińska-Górska [33] and Sosnowska [83]. The authors performed transmission electron microscopy (TEM), scanning electron microscopy (SEM), atomic force microscopy (AFM) visualisations, FTIR, ζ-potential and size distribution, and measured the hydrodynamic diameter in water and size distribution with DLS and Raman measurements. The size of the GO flakes was >5 µm in diameter and up to 1 nm in thickness and the elemental analysis showed that there was >35% carbon and >51% oxygen. The residues were sodium (7%), sulfur (2%), and chlorine (<2%). The FTIR measurements revealed the characteristics for GO bands in the regions of 1649 cm^−1^ and 1700 cm^−1^ [36]. The obtained Raman spectrum was characterized by the occurrence of the following bands: 1359 cm^−1^ (D band), 1602 cm^−1^ (G band), 2720 cm^−1^ (2D band), and 2950 cm^−1^, which were consistent with the reference literature [84]. Oxygen functional groups, such as carboxyl, carbonyl, epoxyl, or hydroxyl, make the GO hydrophilic and are responsible for its adsorbent properties [33].

#### 4.1.2. Cell Lines

The experiment was performed on two normal, adult human skin primary cell lines: human epidermal keratinocytes (HEKa) ATCC-PCS-200-011 and human dermal fibroblasts (HDFa) ATCC-PCS201-012 obtained from the American Type Culture Collection (Manassas, VA, USA). Both lines were cultured on adherent, tissue culture-treated surfaces at standard conditions (temperature of 37 °C, CO_2_ concentration of 5%, and humidity of 91%) in appropriate media, specifically dermal cell basal medium (ATCC-PCS-200-030) supplemented with keratinocyte growth kit (ATCC-PCS-200-040) and fibroblast basal medium (ATCC-PCS-201-030) supplemented with fibroblast growth kit–low serum (ATCC-PCS-201-041).

### 4.2. Methods

#### 4.2.1. Preparation of Nanomaterials and Their Nanocomplexes

The tested solutions of nanomaterials and nanocomplexes were prepared by diluting concentrated nanomaterials in ultrapure water to the desired concentrations. Stock suspensions of D nanoparticles were prepared at a concentration of 1000 mg/mL. A GO solution with a concentration 4000 mg/mL was further diluted in demineralised water to a concentration of 1000 mg/mL. The third suspension consisted of Ag hydrocolloids at a concentration of 50 µg/mL. The stock suspensions were sequentially diluted to working concentrations. The nanocomplexes were obtained by a simple self-assembly after mixing specific volumes of individual nanomaterials at appropriate concentrations. All the suspensions were sonicated using a Vibra-Cell Ultrasonic Processor VCX500 with a High Intensity Cup Horn at 500 W and 20 kHz (Sonics & Materials Inc., Newtown, CT, USA) for 2 min at the amplitude 2.14 µm. The resulting suspensions were added to the culture medium to obtain the working concentrations listed in Table 3. In the case of GOAg, very similar nanocomplexes were analysed by Strojny et al. [36]. FT-IR spectra analysis revealed that the GOAg complex had some of the band characteristics of the single components GO and Ag, but also had new and unique characteristics. The characteristic 1649 cm^−1^ band from GO was present, but the one in the 1700 cm^−1^ region was missing; similarly, the band in the 1686 cm^−1^ region from the Ag was missing. A new band in the spectral region of 1610 cm^−1^, which was not present in the Ag or GO, appeared [36]. This may confirm that the GOAg nanocomplex is not a simple mixture of nanomaterials but a new material of different characteristics.

#### 4.2.2. Morphology and Structure of Nanomaterials

The morphological and structural characteristics of nanomaterials were examined with a transmission electron microscope (TEM). Droplets of sonicated solutions of each kind of nanomaterial and its complex in a low concentration were placed on Formvar-coated 300 mesh Cu grids (Agar Scientific Ltd., Stansted, Essex, UK). The samples were air-dried at room temperature and observed using a JEM-2000EX TEM (JEOL, Akishima, Tokyo, Japan), equipped with a TEM CCD Morada 11-megapixel camera (Olympus Inc., Tokyo, Japan).

#### 4.2.3. ACE2 Inhibition Competitive Test

ACE2 inhibition tests were performed using a COVID-19 Spike-ACE2 binding assay kit II (RayBiotech Life, Peachtree Corners, GA, USA). The test is a rapid, simple, sensitive, and competitive method for characterizing the binding affinity of the S-ACE2 complex in the presence of potential inhibitors, including nanomaterials and their complexes. The kit uses a 96-well plate coated with recombinantly expressed ACE2. The tested nanomaterials were added to the wells in the presence of recombinant S-RBD protein with an Fc tag. Unbound S-RBD was removed through washing and a HRP-conjugated IgG was applied to the wells in the presence of a TMB substrate. The HRP-conjugated IgG binds to the S-RBD protein and reacts with the TMB solution, producing a blue colour with an intensity proportional to the amount of bound S-RBD. The HRP–TMB reaction was halted with the addition of a stop solution, resulting in a blue-to-yellow colour change. The intensity of the yellow colour was then measured as the absorbance at 450 nm in a microplate reader (Infinite M200 TECAN, Tecan i-Control 1.4 software, Zurich, Switzerland). The higher the absorbance, the lower the ability of the tested substance to block the binding. The test results should fall between the absorbance values read for the blank (where neither the S-RBD or nanomaterials were added) and the control (where only S-RBD was added without nanomaterials). The values read for the blank were subtracted from all the other reads for nanomaterials and the control.

#### 4.2.4. In Vitro Cell Treatments

Cell lines were cultured on adherent surfaces in appropriate culture media until they reached a confluence of 80%. Then, working suspensions were added to the culture media at 20% of the final volume (1:5 dilution). The control group (CTRL) for each cell line was cultured in the appropriate medium without the addition of the nanomaterials or nanocomplexes (only ultrapure water at the same volume).

#### 4.2.5. Viability Test

To examine cell viability after incubation with the nanostructures, the XTT test (XTT Cell Proliferation Assay Kit, ATCC, Manassas, VA, USA) was used.

Tetrazolium salts have been widely used as detection reagents for many years in histochemical localization studies and cell biology assays (1,2). XTT is a second-generation tetrazolium dye, which can be effectively utilized in cell proliferation, cytotoxicity, and apoptosis assays (2,3,4). XTT is reduced to a soluble, brightly coloured orange derivative through a combination of cellular effectors. The sensitivity of an XTT assay is greatly improved by using an intermediate electron carrier, N-methyl dibenzopyrazine methyl sulphate (PMS). PMS helps drive XTT reduction and the formation of its formazan derivative.

Cells from the third passage were seeded into a 96-well plate and incubated overnight at 37 °C. The next day, the control medium was removed from the wells and replaced by media with the nanomaterials used in the experiment, which involved adding 100 µL of the medium and 50 µL of the XTT reagent (ATCC, Manassas, VA, USA) to the wells in six replicates. The same media were poured into empty wells to obtain the readings of the blank samples. The plate was incubated for 24 h under standard conditions. Subsequently, the absorbance was measured at a wavelength of 475 nm on a microplate reader (Infinite M200 TECAN, Tecan i-Control 1.4 software, Zurich, Switzerland). Because the nanostructures and media might have their own reducing power, the results for the blank samples (media + nanostructures) were subtracted from the results of the real samples (medium + nanostructures + cells).

#### 4.2.6. Cell Morphology

Photographs showing the morphology of the cells in the culture were taken using an inverted optical microscope (Leica Dmi8, Leica Microsystems GmbH, Wetzlar, Germany) with an installed camera (Leica MC 190 HD, Leica Microsystems GmbH, Wetzlar, Germany).

#### 4.2.7. Statistical Analysis

The data obtained from fluorescence measurements were analysed using one-way analysis of variance (ANOVA), with Statgraphics Plus 4.1 (StatPoint Technologies, Warrenton, VA, USA) and GraphPad Prism ver.10.0.0 (GraphPad Software, Boston, MA, USA). One-way ANOVA was used to determine the influence of the nanostructures on cell viability for each cell line. Additionally, multi-factor ANOVA was used to evaluate the influence of cell line specificity on the response to treatment with nanomaterials and nanocomplexes. To determine whether the differences were statistically significant, a multiple-range test was performed using Fisher’s least significant difference procedure. The results were presented as mean values. Differences of *p* ≤ 0.05 between groups were considered significant.

#### 4.2.8. Viability Coefficients

To compare the changes in viability between groups for the two cell lines, viability coefficients for each line and treatment were used. These coefficients represent cell viability after treatment relative to the cell viability in the control group. Because the viabilities for particular groups were calculated as arithmetic averages, coefficients bore uncertainties resulting from the standard deviations assigned to each mean value. The uncertainty for each coefficient was calculated according to the law of uncertainty propagation [85], using the equation:

$$u\left(y\right)=y\times \sqrt{{\left(\frac{u\left(x_{c}\right)}{x_{c}}\right)}^{2}+{\left(\frac{u\left(x_{t}\right)}{x_{t}}\right)}^{2}}\times k$$

where:

$u\left(y\right)—$expanded standard uncertainty of the viability coefficient;

y—calculated viability coefficient;

$u\left(x_{c}\right)—$uncertainty of the fluorescence measurement for the control group (standard deviation);

$x_{c}—$fluorescence measurement for the control group (mean value);

$u\left(x_{t}\right)—$uncertainty of the fluorescence measurement for the treated group (standard deviation);

$x_{t}—$fluorescence measurement for the treated group (mean value);

k—coverage factor (*k* = 2), defining an interval with a confidence level of 95%.

## 5. Conclusions

Among the investigated nanomaterials, Ag5D10 and Ag5GO10 nanocomplexes seem to be the safest and the most effective combination of nanomaterials for skin application against SARS-CoV-2 infection through ACE2–S binding inhibition. However, to make safe, effective, and efficient application possible, the chosen nanocomplexes should be further investigated using in vivo models, with extended testing and repeated exposure. The expected low specificity of the mentioned nanocomplexes does not exclude the use of them in cases of other viral infections.

## Figures and Tables

**Table 1:** Cell viability coefficients expressing changes after treatment with different concentrations of nanomaterials relative to the control culture ± the expanded standard uncertainty of the viability coefficient (*p* ≥ 95%).

	HEKa	Difference between Cell Lines	HDFa
CTRL	1.00 ± 0.08		1.00 ± 0.04
Ag2	0.77 ± 0.09 *	●	0.93 ± 0.03
Ag5	0.60 ± 0.08 *	●	0.92 ± 0.06
Ag10	0.01 ± 0.01 *	●	0.85 ± 0.06 *
D10	0.94 ± 0.11		1.02 ± 0.08
D20	0.89 ± 0.07		1.00 ± 0.05
D50	0.88 ± 0.10		0.95 ± 0.05
D100	0.86 ± 0.10		0.92 ± 0.08
GO20	0.75 ± 0.10 *	●	0.94 ± 0.08
GO50	0.42 ± 0.07 *	●	0.67 ± 0.04 *
GO100	0.18 ± 0.05 *	●	0.36 ± 0.05 *

*—Viability coefficient significantly differs from a blank in the same cell line; ●—the viability coefficients are significantly different between cell lines.

**Table 2:** Cell viability coefficients expressing viability changes after treatment with nanomaterial complexes relative to the control culture ± expanded standard uncertainty of a viability coefficient, and *p* ≥ 95%.

Group	HEKa	Difference between Cell Lines	HDFa
CTRL	1.00 ± 0.08		1.00 ± 0.04
Ag2	0.77 ± 0.09 *	●	0.93 ± 0.03
Ag2D10	0.78 ± 0.05 *	●	0.89 ± 0.05 *
Ag2D20	0.79 ± 0.10 *		0.90 ± 0.05 *
Ag2D50	0.79 ± 0.12 *		0.84 ± 0.06 *
Ag2GO10	0.83 ± 0.04 *		0.88 ± 0.08
Ag2GO20	0.79 ± 0.07 *		0.90 ± 0.06
Ag2GO50	0.41 ± 0.05 *^Ω^		0.51 ± 0.05 *^Ω^
Ag5	0.60 ± 0.08 *	●	0.92 ± 0.06
Ag5D10	0.80 ± 0.03 *^&^		0.85 ± 0.06 *
Ag5D20	0.71 ± 0.09 *	●	0.90 ± 0.05 *
Ag5D50	0.62 ± 0.09 *	●	0.80 ± 0.05 *^&^
Ag5GO10	0.77 ± 0.13 *		0.84 ± 0.06 *
Ag5GO20	0.56 ± 0.11 *	●	0.83 ± 0.05 *
Ag5GO50	0.34 ± 0.14 *^&^	●	0.55 ±0.04 *^&^

*—The viability coefficient is significantly different from that of the blank; **^Ω^**—the viability coefficient is significantly different from that of Ag2; **^&^**—the viability coefficient is significantly different from that of Ag5; ●—the viability coefficients are significantly different between cell lines.

**Table 3:** Working concentrations of nanomaterials/nanocomplexes in culture media used in experiments on HEKa and HDFa skin cell lines.

Type of Nanomaterial/Nanocomplex	Abbreviation of Applied Treatment	Nanomaterial/Nanocomplex Working Concentration in Culture Medium [µg/mL]
Silver nanoparticles (Ag)	Ag2	2
	Ag5	5
	Ag10	10
Diamond nanoparticles (D)	D10	10
	D20	20
	D50	50
	D100	100
Graphene oxide (GO)	GO10	10
	GO20	20
	GO50	50
	GO100	100
Silver nanoparticles + diamond nanoparticles (AgD)	Ag2D10	2 + 10
	Ag2D20	2 + 20
	Ag2D50	2 + 50
	Ag5D10	5 + 10
	Ag5D20	5 + 20
	Ag5D50	5 + 50
Silver nanoparticles + graphene oxide (AgGO)	Ag2GO10	2 + 10
	Ag2GO20	2 + 20
	Ag2GO50	2 + 50
	Ag5GO10	5 + 10
	Ag5GO20	5 + 20
	Ag5GO50	5 + 50

## Data Availability

Data are contained within the article.
